# Theory and Empiricism in Virulence Evolution

**DOI:** 10.1371/journal.ppat.1004387

**Published:** 2014-10-23

**Authors:** James J. Bull, Adam S. Lauring

**Affiliations:** 1 The Institute for Cellular and Molecular Biology, The University of Texas, Austin, Texas, United States of America; 2 Center for Computational Biology and Bioinformatics, The University of Texas at Austin, Austin, Texas, United States of America; 3 Department of Integrative Biology, The University of Texas at Austin, Austin, Texas, United States of America; 4 Division of Infectious Diseases, Department of Internal Medicine, University of Michigan, Ann Arbor, Michigan, United States of America; 5 Department of Microbiology and Immunology, University of Michigan, Ann Arbor, Michigan, United States of America; University of Florida, United States of America

Dobzhansky famously wrote, “Nothing in biology makes sense except in the light of evolution.” Given the importance of viral evolution to disease emergence, pathogenesis, drug resistance, and vaccine efficacy, it has been well studied by theoreticians and experimentalists. Indeed, as the highly theoretical concepts of quasispecies and error catastrophe gained mainstream attention over the last thirty years, notions of viral populations and viral evolution became almost inseparable for many virologists. In contrast, a large body of theoretical work on the evolution of virulence has yet to gain traction in the virology community. Our purpose here is to offer a brief introduction to virulence theory, explain some of its strengths and weaknesses, and suggest how theory might be united with empiric data. While we focus our discussion on viruses, many of the concepts presented are similarly applicable to other prokaryotic and eukaryotic pathogens.

## What Is Virulence and Does It Evolve?

The generic term “virulence” has many meanings. The fact that empiricists and theorists have different meanings of virulence is not necessarily a problem—understanding the evolution of virulence under any definition would be useful. In the realm of existing theory, it often means mortality—an increased death rate of the infected host. In theoretical models, this narrow framing is convenient; dead hosts do not transmit, so the outcome of virulence has an easily quantified dynamical consequence. Mortality is also universal, and its use as a virulence measure allows for comparative modeling across different systems. However, experimentalists often use sub-lethal measures of virulence, such as weight loss, behavioral change, or damage to a specific organ. As we will show below, such measures are often difficult to incorporate into models of virulence evolution and have led to a gap between theorists and empiricists.

Virulence by most any definition is clearly evolvable; viruses that are serially passaged in laboratory animal experiments will often become more virulent in that host [1]. A “natural experiment” in virulence evolution followed two separate introductions of myxoma virus into rabbit populations in Australia and France in the 1950s. While rabbits infected with this virus initially exhibited mortality rates of >99%, the virus eventually became less virulent [2]. The recent experimental adaptation of H5N1 influenza viruses for respiratory droplet transmission raised fears that increased virulence would accompany selection for transmission [3], [4]. Virulence theory seeks to understand what social and ecological factors drive the evolution of higher and lower virulence, with the hope that predictive models will enable rational virulence management [5].

## How Has the Evolution of Virulence Been Modeled?

**Figure 1:**
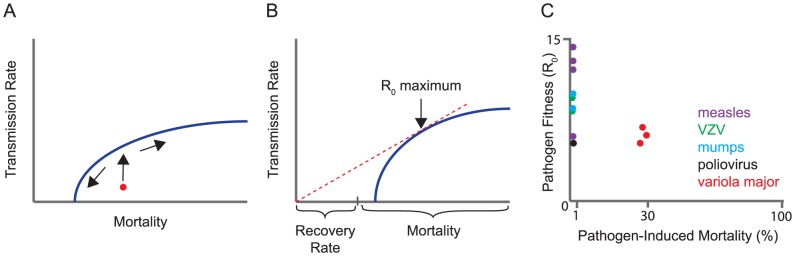
Trade-off models for virulence evolution. (A) A typical trade-off curve for virulence evolution, in this case between transmission rate and host mortality. The trade-off curve is a boundary on the mortality rate and transmission rate that the parasite can evolve. If the characteristics of the pathogen initially lie underneath the trade-off curve (red circle), the early evolution will be toward the boundary, and then along it, shown by arrows. (B) Parasite fitness (R_0_) is proportional to the ratio of transmission rate over the sum of recovery rate and mortality rate. By displacing the trade-off curve along the horizontal axis by an amount equal to the rate of recovery, the R_0_ of any point on the trade-off curve is simply the slope of the line from the origin to the point. The maximum R_0_ is thus achieved at the tangent of a line through the origin, as shown. This figure illustrates how the choice of the trade-off function affects what can be concluded about the evolution of virulence (mortality). Although recovery rate affects the optimum parasite fitness (R_0_), recovery is unaffected by evolution when it is not part of the trade-off (as shown here). However, if the trade-off instead was between transmission and recovery (swapping recovery and mortality rates on the x-axis), evolution of parasite fitness would affect only the rate of host recovery, and there would be no predictions about host mortality. (C) Data on R_0_ and pre-vaccination mortality rates for the viruses indicated (bottom) are abstracted from [16], [17] and references therein. VZV, Varicella zoster virus.

Early theories of virulence suggested that pathogens would evolve to avirulent commensals since harming the host would be a poor long-term survival strategy. This view was challenged in the mid-20th century as evolutionary biologists and population geneticists considered how competition among different strains of a given pathogen would influence the evolution of virulence (see [6] for an excellent historical review). Here, the superiority of one strain over another would depend on its ability to replicate within a host, the length of time that the host is infected (recovery rate), and successful transmission to a new host. These measures of pathogen fitness are easily integrated into a single term, the basic reproductive number (R_0_), which was modeled by Anderson and May [7] as:
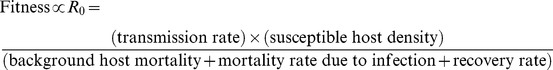
R_0_ gives us a measure of fitness, but not of its evolution. There are several ways to model evolution on this scaffold, and the choice of model is critical. If each parameter in the formula were to evolve independently of the others, a virus could increase its fitness (R_0_) by simply evolving any or all of the following: a lower host mortality rate, a lower recovery rate (longer infectious period), and a higher transmission rate. Instead, most models assume that a subset of these parameters is coupled in a “trade-off.” A trade-off is a genetic constraint that reduces the dimensionality of evolutionary models by forcing one parameter to change with another. Pathogens are assumed to evolve to an optimal balance of these factors subject to the constraints of the trade-off. This balance is often represented graphically as a maximum value on a trade-off curve (Figure 1A, 1B). For example, gains in transmission rate influence virulence by increasing either host mortality or host recovery rates at the population level. Conversely, reducing the length of an infection either by death or pathogen clearance will limit transmission. The most common trade-off, explored in many models, is between transmission and host mortality.

## What Data Support the Existence of a Transmission-Mortality Trade-Off?

Early attempts to validate the transmission-mortality trade-off examined how host mortality limits transmission. Here, the existence of a trade-off can be inferred merely by comparing variants of the same virus that differ in their rates of transmission to see whether mortality also varies—or vice versa. Well-recognized cases in which higher transmission appears to have been linked to higher mortality include feline calicivirus, myxoma virus, H5N2 influenza in avian species, and perhaps smallpox (variola major and variola minor) [2], [8], [9]. Live, attenuated virus vaccines may provide circumstantial evidence for the trade-off model, since they are only rarely transmissible and are much less virulent than their wild-type counterparts. However, the manner in which attenuated vaccines are typically generated makes it difficult to use these data to interpret virulence evolution models. Given the careful work that is required to observe viral variants differing in host mortality and the further difficulty in assessing their relative transmission rates, we know little about the shape of the trade-off curves and the location of the maxima for these viruses. Thus, it is certainly plausible that the paucity of documented trade-off variants is due to insufficient observations rather than their absence.

## Is the Transmission-Mortality Trade-Off Broadly Applicable?

Whereas some evidence supports a transmission-mortality trade-off, other observations do not. The most straightforward interpretation of models for the evolution of fitness suggest that a pathogen's R_0_ at an evolutionary equilibrium would entail at least a modest level of disease-induced mortality (see arrow, Figure 1B). The reasoning is that if host mortality is very low, the denominator of R_0_ is dominated by the recovery rate. In a transmission-mortality trade-off, evolution should proceed until further gains in transmission are offset by increases in mortality. For a large number of infectious diseases, including many common and highly transmissible human viral infections, the case fatality rate is indeed very low, 0.001 to 0.01 (Figure 1C). Although the case fatality rate does not strictly coincide with the mortality rate in the R_0_ formula above, in these cases a low case fatality rate implies a low mortality rate. Modest increases in transmission should be possible and almost unconditionally beneficial to pathogen fitness, and yet have not been observed. Rather, the relatively high fitness (R_0_) and low mortality rate for many viruses suggests that a factor other than host mortality is limiting further transmission gains. It is also difficult to apply the trade-off model to many viral infections in which the majority of individuals are asymptomatic and yet efficiently shed virus. On balance, it is hard to reconcile low case-fatality rates of many human viruses with the main prediction of the trade-off model—that there is an optimum at which viral transmission is offset by host mortality.

## If Host Mortality Is Frequently Not the Factor Limiting Higher Transmission Rates, What Is?

The principle that natural selection on infectious agents will favor between-host transmission seems well founded, and nearly everything in evolution involves a trade-off. The choice of which trade-off function to use (e.g., transmission-mortality or transmission-recovery) is thus absolutely critical to understanding the evolution of virulence; yet, we have little empirical understanding of the trade-offs involved. While it made for more quantitative and precise models, the early focus on host mortality obscured the importance of recovery rate and other sublethal measures of virulence as limiting factors for transmission. In sublethal infections, host control will place a boundary on viral replication, which will tend to reduce the length of an infection (increasing recovery rate), and therefore limit transmission. Given the diversity in “life history” among viruses, we suspect that there will be many viral and host factors at play with one or several being limiting for a given agent.

### Intrinsic limits on pathogen replication and spread

Trade-off models assume that, aside from the trade-off, a virus can evolve to infinite extremes, from arbitrarily low levels of transmission to arbitrarily high ones. Yet viral evolution is bounded; there must be a maximal rate of viral entry, replication, assembly, and spread within a given tissue or host. A virus near this bound cannot do any better—it will not be able to evolve higher transmission or virulence, even if selection favors an increase. We know little about predicting these evolutionary boundaries, but experimental adaptations commonly reveal their existence [10].

### Host immunity

Invading pathogens are rapidly sensed by the immune system, and inducible immune effectors are perhaps the most significant barriers to intra- and inter-host spread. Both innate and adaptive immune responses will clearly limit viral replication and transmission. Peak viral loads—and transmission—are often observed just prior to the onset of symptoms, a surrogate marker for the inflammatory response. Further evidence for the importance of immune control as a rate-limiting factor in transmission is the prolonged, often asymptomatic, shedding of viruses in immunodeficient hosts. The relationship between viral replication, immunity, and pathogenesis is clearly a complex one, because stronger immune responses will limit the transmission of some viruses and increase the immunopathologic manifestations, or virulence, of others. Of the evolutionary models that incorporate host immunity as a limiting factor, several invoke trade-offs [11].

## How Can Empiric Data Lead to Better Models of Virulence Evolution?

While the transmission-mortality trade-off perhaps applies to a subset of pathogens, the complex intra-host dynamics of many infectious diseases make it poorly generalizable. We believe that more empiric work is needed on the relationships between transmission, virulence, and recovery rate. These data will define the mechanistic nature of the trade-offs, if any, that are specific to a given pathogen and will ultimately lead to better models. For example, population-level studies of chronic human immunodeficiency virus infection suggest that intermediate viral loads maximize transmission potential, reflecting a potential trade-off between the transmission and the duration of asymptomatic infection [12]. There is also a clear need for comparative analysis of transmission and virulence among strains of a given pathogen. In the H5N1 influenza system, one could use ancestral and evolved strains to examine how virulence was affected by selection for increased airborne transmission [3], [4]. Experimental work may also elucidate how heterogeneity in host immune function influences the evolution of virulence and transmission [13]. Finally, the virology literature is replete with studies of interactions between virus and cell. This type of work would go far toward elucidating the evolution of virulence if those studies also addressed the likely consequences of virus–host dynamics for transmission. More refined datasets will enable heavily parameterized, multiscale computational models that describe cell–cell and tissue-specific viral transit, ultimately leading to viral release from the host [14], [15]. The time is ripe to bring this new dimension of evolutionary virology into the fold, and models based on empiric data will allow for better identification and control of emerging and rapidly evolving pathogens.
